# Characteristics of Sleep Disturbance in Patients with Long COVID: A Retrospective Observational Study in Japan

**DOI:** 10.3390/jcm11247332

**Published:** 2022-12-09

**Authors:** Naruhiko Sunada, Yasuhiro Nakano, Yuki Otsuka, Kazuki Tokumasu, Hiroyuki Honda, Yasue Sakurada, Yui Matsuda, Toru Hasegawa, Daisuke Omura, Kanako Ochi, Hideharu Hagiya, Keigo Ueda, Hitomi Kataoka, Fumio Otsuka

**Affiliations:** 1 Department of General Medicine, Okayama University Graduate School of Medicine, Dentistry and Pharmaceutical Sciences, Okayama 700-8558, Japan; 2 Center for Education in Medicine and Health Sciences, Okayama University Graduate School of Medicine, Dentistry and Pharmaceutical Sciences, Okayama 700-8558, Japan

**Keywords:** insomnia, myalgic encephalomyelitis, chronic fatigue, long COVID, Omicron variant, post COVID-19 condition

## Abstract

Objectives: The objective of this study was to determine the clinical and endocrinological features of sleep disturbance in patients with long COVID. Methods: This study was a single-center retrospective observational study for patients who visited the COVID-19 aftercare outpatient clinic (CAC) established in Okayama University Hospital in Japan during the period from 15 February 2021 to 29 July 2022. The long COVID patients were divided into two groups based on the presence or absence of sleep disturbance, and the clinical and laboratory characteristics of the patients were analyzed. Results: Out of 363 patients with long COVID, after excluding 6 patients, 60 patients (16.5%) (55% males, median age of 38 years) complaining of sleep disturbance were compared with 303 patients (83.5%) (43% males, median age of 40 years) without sleep-related symptoms. Although there were no significant differences in clinical backgrounds and severities of COVID-19 between the two groups by the multivariate analysis, the percentage of long COVID patients with sleep disturbance was significantly increased among patients infected in the Omicron-dominant phase. In addition, the prevalence rate of sleep disturbance in patients when infected in the Omicron phase (24.8%) was two-times higher than that in patients infected in the Delta phase (12.8%). Of note, the percentages of patients with sleep disturbance who also complained of general fatigue, headache, concentration loss, anxiety, low-grade fever, and brain fog symptoms were higher than the percentages of patients without sleep disturbance who had the same complaints. Among the types of sleep disturbance, the percentage of patients who complained of loss of sleep induction (75%) was much higher than the percentage of patients with early-awakening sleep disturbance (6.7%), and many of the patients with mid-awakening types of insomnia had brain fog symptoms. Endocrine examinations revealed that long COVID patients with sleep disturbance had significantly higher levels of plasma adrenocorticotropin and lower levels of serum growth hormone, suggesting the presence of hypothalamic–pituitary stress. Conclusion: The prevalence of sleep disturbance has been increasing in long COVID patients infected in the Omicron phase with a certain clinical and endocrine trend.

## 1. Introduction

It has been almost three years since the declaration of a coronavirus disease 2019 (COVID-19) pandemic by the World Health Organization (WHO), and the cumulative number of infected people worldwide exceeded 600 million, including 20 million in Japan, as of September 2022 (https://covid19.who.int/table, accessed on 23 September 2022). COVID-19 also causes prolonged symptoms called either long COVID, post-acute sequelae of severe acute respiratory syndrome coronavirus 2 (SARS-CoV-2) (PASC), or post COVID-19 condition (PCC) [1]. Such prolonged conditions have been experienced in approximately one-third of COVID-19 patients, even a few months after the acute phase of infection [2,3]. In a recent cohort study in the Netherlands, it was found that persistent symptoms attributed to COVID-19 occurred in 12.7% of infected patients by considering the pre-infection conditions and the symptom dynamics of uninfected controls [4].

A systematic review of post-acute symptoms of COVID-19 [5] showed that there were more than thirty symptoms and signs in post-acute COVID-19. The common manifestations were fatigue, dyspnea, alopecia, hyperhidrosis, insomnia, anxiety, and arthralgia, and insomnia had the second-highest frequency (26%) [5]. Another systematic review [6] that included information from 32 countries showed that there was an increase in the prevalence of mental health issues in the general population during the COVID-19 pandemic, with estimated global prevalence of depression, anxiety, and sleep problems of 27% to 28%, indicating that social and economic burdens during and after epidemics or pandemics have a great global impact. Among these frequent symptoms of long COVID, insomnia has recently been recognized as a neuropsychological disorder leading to various medical issues including cognitive impairments, emotional distress, negative thoughts, and further medical disorders [7]. In addition, sleep disturbance has been reported in various populations, not only in the acute phase of infection, but also in the chronic phase after recovery from acute symptoms [8].

However, the characteristics of long COVID patients with sleep disturbance have yet to be elucidated. We have established a COVID-19 aftercare outpatient clinic (CAC) and reported the clinical characteristics of long COVID patients. It was found that the major complaints in the early period of the COVID-19 pandemic were fatigue and anxiety [9] and that the severity of the infection status affected the number and duration of persistent symptoms [10]. The frequency of each symptom of long COVID has been changing with the emergence of new variants of SARS-CoV-2 [11]. Moreover, we have shown that endocrine factors including anterior pituitary, adrenal and thyroid hormones [12], and male androgen levels [13,14] might be related to the fatigue symptoms of long COVID.

The aim of the present study was to determine the characteristics of sleep disturbance in patients with long COVID by focusing on the clinical backgrounds and endocrine data and to clarify the changes in clinical manifestations in long COVID patients who were infected in the different phases.

## 2. Methods

### 2.1. Study Design and Inclusion of Patients

This study was a retrospective observational study conducted in a single hospital. We reviewed medical records of patients who visited our CAC during the period from 15 February 2021 to 29 July 2022. Long COVID was defined as symptoms that persist for more than four weeks after the onset of COVID-19. We obtained information on age, sex, body mass index (BMI), current habits of smoking and drinking, COVID-19-related hospitalization, therapeutic use of corticosteroids or oxygen in the acute phase, severity of COVID-19, history of COVID-19 vaccination, number of days between onset of COVID-19 and first CAC visit, and clinical symptoms of PCC. The severity of the acute phase of COVID-19 was classified according to the criteria defined by the Ministry of Health, Labour and Welfare in Japan [15].

Clinical symptoms of long COVID were identified through a physician’s face-to-face careful medical interview. Based on individual interviews, long COVID patients whose chief complaints involved insomnia were classified as patients having a sleep disturbance. Based on the patients’ subjective complaints, sleep disturbances were further separated into four types including disturbed sleep induction, mid-awakening, decreased deep sleep, and early-awakening types of insomnia. The presence of “brain fog” symptoms was carefully determined by individual interview, based on complaints of the subjective feeling of concentration difficulty and impaired ability to concentrate and/or think; namely, being mentally slow, fuzzy, or spaced out, affecting the patient’s ability to think or concentrate with a dull headache [16]. The onset of COVID-19 in the patients was divided into three groups based on the epidemiological aspects of COVID-19 in Okayama Prefecture in Japan: preceding period; Delta-dominant period; and Omicron-dominant period [11]. The preceding period is the period from the ancestral strain to the Alpha strain, before 18 July 2021; the Delta-dominant period is the period from 19 July 2021 to 31 December 2021, when the Delta variants were dominant after the Alpha-dominant phase; and the Omicron-dominant period is the period after 1 January 2022, when the Omicron variants were dominant in Okayama Prefecture, Japan.

### 2.2. Endocrine Examination

The examinations of circulating hormones were determined by each physician for evaluating various endocrine disorders. Blood collections were carefully performed in a relaxed sitting position around late morning to early afternoon time in the same way when the patients visited our clinic. The auto-analyzer system Cobas 8000 (F. Hoffmann-La Roche AG, Basel, Switzerland) was used for measuring hormone levels at the Central Laboratory of our facility. As reported previously [12], assays for plasma adrenocorticotropin (ACTH) and serum cortisol were performed by an electro-chemiluminescence immunoassay (ECLIA) using Elecsys ACTH and Elecsys Cortisol II kits (F. Hoffmann-La Roche AG), respectively. The assays for serum free-thyroxin (FT4) and thyrotropin (TSH) were performed by Elecsys FT4 III and TSH kits, and the assays for serum GH and IGF-I were completed by Elecsys GH and IGF-I kits (F. Hoffmann-La Roche AG), respectively. Serum levels of IGF-I were then calculated to the values of standard deviation (SD) [17].

### 2.3. Statistical Analysis

For all the statistics, EZR, version 1.40 (Saitama Medical Center, Jichi Medical University, Saitama, Japan), a graphical user interface for R (The R Foundation for Statistical Computing, Vienna, Austria), was utilized [18]. This system is modified from R commander, including frequently used functions in the biostatistical analysis. To compare the clinical backgrounds of patients with, and those without, sleep disturbance, the percentages of patients with sleep disturbance in the three variant phases, the percentages of major symptoms accompanied by, and not accompanied by, sleep disturbance, the percentages of patients with the four types of insomnia, and the hormonal characteristics of patients with, and those without, sleep disturbance, the Mann–Whitney U test and Pearson’s χ^2^ test were performed for non-normal distributed variables and for categorical variables, respectively. In addition, to investigate an associating factor for the sleep disturbance, we incorporated six factors (gender, smoking habit, alcohol drinking habit, severity (moderate and severe cases versus mild cases), infected periods (Omicron phase versus others), and vaccination (2 doses or more versus 1 dose or less)) into the logistic regression model. The thresholds for statistical significance were defined as * *p* < 0.05 and ** *p* < 0.01.

### 2.4. Ethical Approval

This study was approved by the Ethics Committee of Okayama University Hospital (No. 2105-030) and adhered to the Declaration of Helsinki. Information on the protocol of this study was provided on the website of our hospital and on the clinic wall in our hospital. Because of the anonymization of the patients’ data, informed consent from the patients was not necessary, and the patients who wished to opt out were offered the opportunity.

## 3. Results

Data for the 369 patients who visited our CAC during the study period were obtained from medical records. We excluded six patients, including two patients who visited our CAC within four weeks after onset of COVID-19, two patients who were under ten years of age, and two patients who had insufficient available data. The clinical backgrounds of the remaining 363 patients are shown in Table 1. The eligible cases were classified into two groups: one group with complaints of sleep disturbance (60 patients) and one group without complaints of sleep disturbance (303 patients). There were no significant differences between the two groups in age, gender, BMI, smoking and alcohol habits, and number of days from infection to the first visit to the CAC. COVID-19 acute phase conditions, including the hospital admission rate, the percentage of patients who received oxygen and/or steroid therapy, and the severity of COVID-19, were also not significantly different between the two groups. The percentage of patients suffering from sleep disturbance was significantly higher in patients infected in the Omicron-dominant period than those infected in the preceding phase and the Delta-dominant phase. Histories of COVID-19 vaccination and the doses did not affect the prevalence of sleep disturbance in the symptoms of long COVID (Table 1).

**Figure 1:**
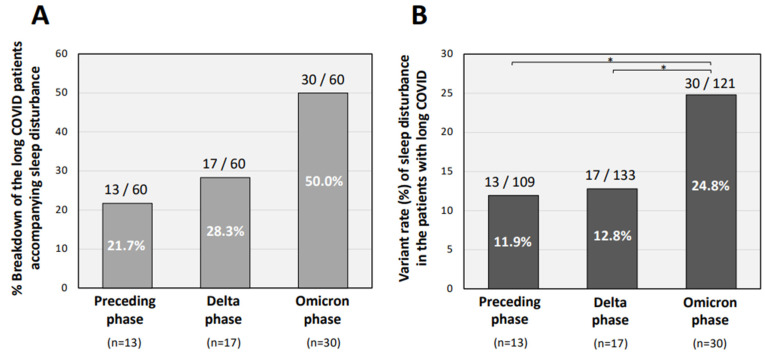
Percentage of patients with sleep disturbance in long COVID patients infected in each variant phase. (**A**) The percentage of long COVID patients with sleep disturbance in patients infected in each variant-based phase is shown. (**B**) Frequency of sleep disturbance in long COVID patients infected in each variant phase is shown. The preceding period was the period from the ancestral strain to the Alpha strain, before 18 July 2021; the Delta phase was the period from 19 July 2021 to 31 December 2021, when the Delta variants were dominant after the Alpha-dominant phase; and the Omicron phase was the period after 1 January 2022, when the Omicron variants were dominant in Okayama Prefecture, Japan. The χ^2^ test was performed for each symptom between the groups and * *p* < 0.05 was considered as statistically significant.

The percentages of patients with sleep disturbance at the first CAC visit in the three groups of viral variant phases are shown in Table 1 and Figure 1. The percentage of long COVID patients with sleep disturbance was significantly higher in the patients infected in the Omicron-dominant phase (30 of 60 patients: 50.0%) than in the patients infected in the Delta-dominant phase (17 of 60 patients: 28.3%) and patients infected in the preceding phase (13 of 60 patients: 21.7%; Figure 1A). The incidence rate of sleep disturbance was, significantly, two-times higher in patients in the Omicron-dominant phase (30 of 121 patients: 24.8%) than those in the Delta-dominant phase (17 of 133 patients: 12.8%), and those in the preceding phase (13 of 109 patients: 11.9%; Figure 1B). However, as a result of the multivariate analysis, all the factors included were revealed to be not related with the sleep disturbance (Table 2).

**Figure 2:**
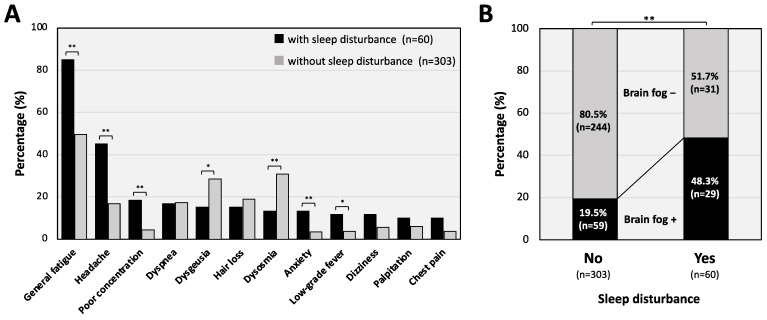
Major symptoms accompanying sleep disturbance in patients with long COVID. (**A**) Percentages of patients with major long COVID symptoms in long COVID patients with and those without sleep disturbance. (**B**) Prevalence of brain fog symptoms in long COVID patients with, and those without, sleep disturbance. The χ^2^ test was performed for each symptom between the groups and * *p* < 0.05 and ** *p* < 0.01 were considered as statistically significant.

The presence of other comorbidity symptoms accompanying sleep disturbance was also analyzed (Figure 2). Of note, the percentages of patients with sleep disturbance who also complained of general fatigue (85%), headache (45%), concentration loss (18.3%), anxiety (13.3%), and low-grade fever (11.7%) were significantly higher than the control patient without the sleep disturbance (Figure 2A). Among severe fatigue and neuropsychiatric symptoms, impairment in cognitive functions has been recognized as a sort of syndrome of “brain fog” [19], which is also related to sleep disturbance. Of interest, brain fog symptoms were significantly more frequent in the patients with sleep disturbance (48.3%) than in those without sleep disturbance (19.5%; Figure 2B).

**Figure 3:**
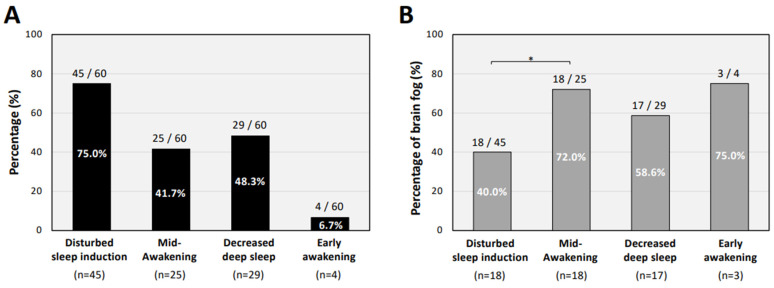
Types of insomnia in long COVID patients with sleep disturbance. (**A**) Four types of sleep disturbance, including disturbed sleep induction, mid-awakening, decreased deep sleep, and early awaking in long COVID patients with insomnia. (**B**) Prevalence of brain fog symptoms in long COVID patients with each type of insomnia. The χ^2^ test was performed for each symptom between the groups and * *p* < 0.05 was considered as statistically significant.

Sleep disturbance was further divided into four types of insomnia (Figure 3). Among the four types of sleep disturbance, the percentage of patients with loss of sleep induction (45 of 60 patients: 75%) was much higher than the percentage of patients with an early-awakening condition (4 of 60 patients: 6.7%; Figure 3A). Brain fog symptoms were significantly more frequent in patients with mid-awakening insomnia (72%) than those with disturbed sleep induction (40%; Figure 3B).

**Figure 4:**
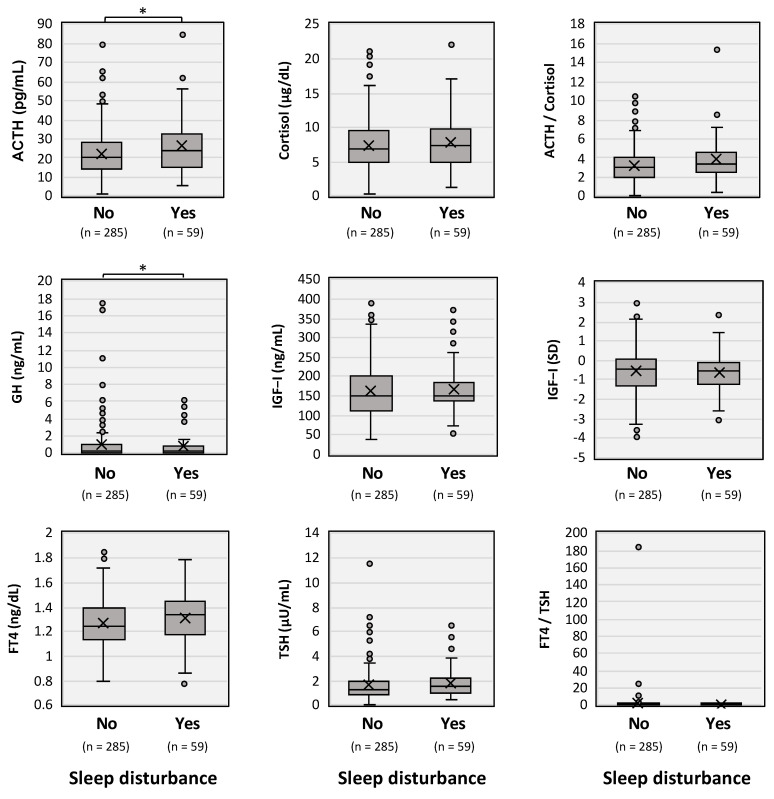
Hormonal trends in long COVID patients with sleep disturbance and those without sleep disturbance. Circulating levels of adrenocorticotropin (ACTH), cortisol, growth hormone (GH), insulin-like growth factor (IGF)-I, free thyroxin (FT4), and thyrotropin (TSH) were shown on the long COVID patients with (Yes) and those without (No) sleep disturbance. The upper horizontal line of the box: the 75th percentile; the lower horizontal line of the box: the 25th percentile; the horizontal bar within the box: the median; the upper horizontal bar outside the box: the maximum value within 1.5-times the interquartile range; and the lower horizontal bar outside the box: the minimum value within 1.5-times the interquartile range. The Mann–Whitney U test was performed and * *p* < 0.05 indicated a statistically significant difference between the indicated groups.

Moreover, as shown in Figure 4, the results of endocrine examinations obtained from 344 out of 369 patients revealed that long COVID patients with sleep disturbance (59 patients) had significantly higher levels of plasma ACTH and lower levels of serum GH than those in patients without sleep disturbance (285 patients). On the other hand, serum FT4 levels tended to be slightly, but not significantly (*p* = 0.0552), higher in the long COVID patients with sleep disturbance. Serum levels of cortisol, IGF-I, and TSH, and the ratios of ACTH/cortisol, and FT4/TSH were not significantly different between the two groups.

## 4. Discussion

The aim of this study was to determine the clinical characteristics of sleep disorders in patients with long COVID based on data obtained in a single-center retrospective observational study for patients who visited our hospital. Of 363 patients with long COVID who visited our outpatient clinic, 60 patients (16.5%) complained of sleep disturbance. Results of previous studies have shown that the proportions of COVID-19 patients with sleep disturbances during the pandemic ranged from 11% to 75% [5,6,20,21,22,23]. A systematic review showed that the prevalence of sleep problems caused by the COVID-19 pandemic has been as high as 40% of the general population, and health care workers have also been affected by sleep problems [20]. Although there have been several reports related to sleep disturbances after COVID-19, our study is quite unique in that the clinical characteristics of sleep disturbance among the long COVID symptoms related to the viral variants were evaluated.

Of note, the proportion of long COVID patients with sleep disturbance was 50% among patients infected in the Omicron-dominant phase, although there were no significant differences in the multivariate analysis adjusting for other factors. The prevalence of sleep disturbance was two-times higher in patients infected in the Omicron phase (24.8%) than in patients infected in the Delta phase (12.8%), suggesting that sleep disturbance is one of the characteristic symptoms of long COVID in patients infected in the Omicron-dominant phase. A prospective observational study showed that the patients with COVID-19 in the acute phase who were infected during the period of Omicron variant dominance had less frequent loss of smell, more frequent sore throat, and a lower rate of hospitalization than the patients who were infected during the period of Delta variant dominance [24,25], suggesting that the Omicron and Delta variants have different features in the acute phase as well as the chronic phase. Another case-control observational study showed that the incidence of long COVID at 28 days after diagnosis of COVID-19 in COVID-19 patients who were infected during the Omicron-dominant period (4.5%) was less than half of that in COVID-19 patients who were infected during the Delta-dominant period (10.8%) [26]. On the other hand, the results of retrospective studies in patients with COVID-19 for 2 years showed increased risks of psychotic disorders, cognitive damage, dementia, and epilepsy, and that the neuropsychiatric outcomes were almost similar in the Delta- and Omicron-dominant phases [27].

In the present study, many long COVID patients with sleep disturbance complained of other symptoms including general fatigue, headache, concentration loss, anxiety, low-grade fever, and brain fog symptoms. The results of this study also revealed that the proportions of patients with sleep disturbance who complained of general fatigue and brain fog symptoms were higher than the proportions of patients without sleep-related symptoms who had the same complaints. Among the types of sleep disturbance, loss of sleep induction was the most frequent (in 75% of the patients). Clinically, insomnia has been considered as one of the major psychiatric disorders, with the main symptoms being perceived sleep dissatisfaction and difficulty in initiating or maintaining sleep [28,29]; additionally, the incidence is higher in young adults as shown in the present study in which the median age of patients with sleep disturbance was 38 years. The present results regarding the types of sleep disturbance provide possible clues for selecting prescriptions of sleep inducers for patients with insomnia related to long COVID.

We also found that the proportion of long COVID patients with loss of sleep induction (75%) was higher than the proportion of patients with early-awakening types of insomnia (6.7%). Of interest, brain fog symptoms were more frequent in patients with mid-awakening (72%) types of insomnia than in patients with sleep induction failure (40%) in the present study. Such a clinical condition complicated with sleep disorders and brain fog may arise from a combined mechanism of biological and psychological damages [19]. A persistent systemic inflammatory state [30], in addition to social isolation and trauma during acute infection and chronic fatigue [31], might be complexly and individually involved in the neuropsychiatric symptoms, especially in patients with sleep disturbances.

In our earlier study on fatigue due to long COVID, the prevalence rate of myalgic encephalomyelitis/chronic fatigue syndrome (ME/CFS) was calculated to be 16.8%, based on data for 279 patients with long COVID [32]. ME/CFS is characterized not only by fatigue, but also by sleep disturbance, and various types of pain and neurological/cognitive dysfunction, and persists for more than six months [32]. The increase in long COVID patients suffering from fatigue with insomnia in the Omicron phase suggests that attention should be paid to the possibility of development of ME/CFS in long COVID patients complaining of sleep disorders.

It was also revealed that patients with sleep disturbance had significantly higher levels of plasma ACTH but lower levels of serum GH, suggesting that increased stress on the hypothalamic–pituitary axis accelerated corticotrope activity but suppressed somatotropin function in the pituitary. In our earlier study on hormonal data for long COVID patients [12], fatigue and depression scores were found to be correlated with serum levels of FT4 and cortisol, respectively, and patients complaining of general fatigue showed lower levels of serum GH. Considering the results of the present study, showing that long COVID patients had significantly higher levels of ACTH but lower GH secretion, long COVID may be linked to functional alteration of the hypothalamic–pituitary axis under the condition of prolonged stress and its resilience [33].

As for the relationships of endocrinological and neurological aspects, COVID-19 seems to affect the hypothalamic–pituitary–adrenal (HPA) axis, which is stimulated by various stresses and can influence the emotional conditions in patients with COVID-19 [34]. Microglia, immune effector cells in the central nervous system, express corticotropin-releasing hormone (CRH) receptors that might be activated by the stress caused by COVID-19 [34]. Given that microglia interact with mast cells in the brain leading to neuroinflammation, activation of the hypothalamic microglia might lead to cognitive dysfunction and brain fog symptoms, as seen in cases of mast cell activation syndrome [34].

The present study has the following limitations. First, since this study was a single-center retrospective study conducted in Japan, there are limitations on the data that can be obtained by medical records. A multicenter prospective study would be ideal to examine the entire population of long COVID patients. Second, since blood sampling for hormonal evaluation was performed only once when the patients visited our outpatient clinic, circadian changes associated with sleep disturbance could not be taken into account. Third, we classified infections with the Omicron and Delta variants on the basis of the prevalence period, not by genetic testing of the strains individually. Fourth, the precise intervals after vaccinations were not considered when the number of vaccinations was examined. Fifth, discrepancies in the definitions of cognitive dysfunctions such as brain fog may limit the accuracy of estimation of each related manifestation. A standardized definition of brain fog using quantitative neurological tests and sleep-pattern analysis using polysomnography would be needed in future studies. Sixth, we only counted the patients’ subjective complaints of insomnia as sleep disturbance, which might have underestimated milder symptoms of sleep disturbance being masked by other more prominent symptoms.

Collectively, we uncovered that 16.5% of total patients with long COVID, reaching up to half of those in the Omicron-dominant phase, complained of sleep disturbance. Three-fourths of the patients with insomnia suffered from sleep induction failure, and the presence of the insomnia appeared to be associated with the fatigue-related symptoms of long COVID, such as headache, poor concentration, anxiety, and brain fog. Notably, our endocrine data indicated that these manifestations could be associated with increased stress of the hypothalamic–pituitary axis. Further study would be necessary to clarify the biological underlying mechanism for the increased frequency of sleep disturbance in long COVID patients in the Omicron-dominant phase.

## Figures and Tables

**Table 1:** Backgrounds of long COVID patients with, and those without, sleep disturbance.

		Sleep Disturbance		*p* Value
Sleep disturbance, *n* (%)		No: 303 (83.5%)	Yes: 60 (16.5%)	
Age, median (IQR)		40 (26.5–50)	38 (22.75–49.5)	0.504
Gender, *n* (%)		Male 131 (43.2%) Female 172 (56.8%)	Male 33 (55%) Female 27 (45%)	0.126
BMI, median (IQR)		22.9 (20.4–26.0)	23.5 (20.4–26.8)	0.948
Smoking habit, *n* (%)		145 (47.9%)	26 (43.3%)	0.617
Alcohol-drinking habit, *n* (%)		109 (36.0%)	26 (43.3%)	0.352
Days after onset to first visit, median (IQR)		88 (55.5–131.5)	71.5 (50.75–102.75)	0.0698
COVID-19-related clinical background				
Admission, *n* (%)		80 (26.4%)	13 (21.7%)	0.545
Steroid and/or O_2_ therapy, *n* (%)		43 (14.2%)	9 (15%)	1.00
Severity, *n* (%)	Mild	236 (77.9%)	52 (86.7%)	0.174
	Moderate/Severe	67 (22.1%)	8 (13.3%)	
Infected periods, *n* (%)	Preceding	96 (31.7%)	13 (21.7%)	0.164
	Delta-dominant	116 (38.3%)	17 (28.3%)	0.189
	Omicron-dominant	91 (30.0%)	30 (50.0%)	** <0.01
Vaccinations, *n* (%)	None	136 (44.9%)	25 (41.7%)	0.752
	2 doses or more	138 (45.5%)	30 (50.0%)	0.572
	Unknown	5 (1.65%)	1 (1.67%)	1.00

Medians [IQR: interquartile ranges] and percentages (%) are shown. BMI: body mass index; COVID-19: coronavirus disease 2019. The Mann–Whitney U test and χ^2^ test were performed when appropriate for statistics, and ** *p* < 0.01 indicates statistically significant differences.

**Table 2:** Multivariate analysis to investigate factors associated with the sleep disturbance.

	Odds Ratio (95% Confidence Interval)	*p* Value
Gender (male)	1.58 (0.87–2.86)	0.13
Smoking habit	0.70 (0.38–1.30)	0.26
Alcohol-drinking habit	1.52 (0.83–2.80)	0.17
Severity (moderate and severe)	0.41 (0.14–1.17)	0.09
Infected periods (Omicron phase)	1.76 (0.62–4.97)	0.29
Vaccination (2 doses or more)	0.90 (0.57–1.41)	0.64

A multivariate logistic regression model was used to identify variables associated with sleep disturbance.

## Data Availability

Detailed data will be available if requested from the corresponding author.
